# Factors associated with higher healthcare costs in a cohort of homeless adults with a mental illness and a general cohort of adults with a history of homelessness

**DOI:** 10.1186/s12913-021-06562-6

**Published:** 2021-06-06

**Authors:** Kathryn Wiens, Laura C. Rosella, Paul Kurdyak, Simon Chen, Tim Aubry, Vicky Stergiopoulos, Stephen W. Hwang

**Affiliations:** 1 Dalla Lana School of Public Health, University of Toronto, 155 College St., Toronto, Ontario M5T 1P8 Canada; 2 Centre for Addiction and Mental Health, 33 Russel St, Toronto, Ontario M5S 3M1 Canada; 3 ICES, 2075 Bayview Avenue, Toronto, Ontario M4N 3M5 Canada; 4 School of Psychology & Centre for Research on Educational and Community Services, University of Ottawa, 136 Jean-Jacques-Lussier Private, Ottawa, Ontario K1N 9A8 Canada; 5 MAP Centre for Urban Health Solutions, St. Michael’s Hospital, 30 Bond St, Toronto, Ontario M5B 1X1 Canada

**Keywords:** Homeless, Healthcare, Costs

## Abstract

**Background:**

Healthcare costs are disproportionately incurred by a relatively small group of people often described as high-cost users. Understanding the factors associated with high-cost use of health services among people experiencing homelessness could help guide service planning.

**Methods:**

Survey data from a general cohort of adults with a history of homelessness and a cohort of homeless adults with mental illness were linked with administrative healthcare records in Ontario, Canada. Total costs were calculated using a validated costing algorithm and categorized based on population cut points for the top 5%, top 6–10%, top 11–50% and bottom 50% of users in Ontario. Multinomial logistic regression was used to identify the predisposing, enabling, and need factors associated with higher healthcare costs (with bottom 50% as the reference).

**Results:**

Sixteen percent of the general homeless cohort and 30% percent of the cohort with a mental illness were in the top 5% of healthcare users in Ontario. Most healthcare costs for the top 5% of users were attributed to emergency department and inpatient service costs, while the costs from other strata were mostly for physician services, hospital outpatient clinics, and medications. The odds of being within the top 5% of users were higher for people who reported female gender, a regular medical doctor, past year acute service use, poor perceived general health and two or more diagnosed chronic conditions, and were lower for Black participants and other racialized groups. Older age was not consistently associated with higher cost use; the odds of being in the top 5% were highest for 35-to-49-year year age group in the cohort with a mental illness and similar for the 35–49 and ≥ 50-year age groups in the general homeless cohort.

**Conclusions:**

This study combines survey and administrative data from two cohorts of homeless adults to describe the distribution of healthcare costs and identify factors associated with higher cost use. These findings can inform the development of targeted interventions to improve healthcare delivery and support for people experiencing homelessness.

**Supplementary Information:**

The online version contains supplementary material available at 10.1186/s12913-021-06562-6.

## Background

The majority of healthcare costs in the United States and Canada are attributed to the top 5% of healthcare users, a group of people often referred to as “high-cost users” [1]. End-of-life care contributes to elevated healthcare costs; however, a recent study reported that less than one-third of high-cost users are in their final year of life [2]. In addition, complex physical and mental health conditions, along with other social factors such as homelessness, contribute to a consistent need for costly health services throughout the life course [3].

Administrative healthcare data sources are commonly used to study high-cost users [4–7]; however, these data only capture few individual level characteristics due to limited documentation in medical charts. Data linkage can overcome this limitation by combining healthcare cost data with survey data. Recently, a set of studies linked a housed cohort of participants enrolled in the Canadian Community Health Survey with administrative health records in Ontario to examine factors associated with higher cost use [5, 8]. Given the detrimental impacts of homelessness on health – notably the high prevalence of chronic disease complications, infectious conditions, and violence-related injury [9, 10], along with competing priorities of food and shelter needs [11] – it is also necessary to examine factors associated with higher costs among people experiencing homelessness to inform service delivery.

While access to housing remains a priority, a more thorough understanding of the distribution of healthcare costs can inform hospital service planning and resource allocation. Further, identifying the individual-level characteristics associated with higher cost use can inform the development of tailored interventions to support homeless patients and strategies to target upstream factors and reduce avoidable costs to the system [1, 5].

Most research on healthcare utilization within homeless populations focused on frequent use of the emergency department (ED) [12, 13]. The focus on a single type of healthcare encounter does not capture complete patterns of use. In comparison, healthcare cost data is a useful composite measure that combines the frequency and intensity of health service utilization. Using a validated costing algorithm to estimate individual-level costs, this study describes the distribution of healthcare costs by adults experiencing homelessness and identifies factors that are associated with higher cost use in the following year using cost gradient categories from the general Ontario population (top 5%, top 6–10%, top 11–50%, bottom 50% of users). These associations were examined in two cohorts of adults experiencing homelessness in Ontario: a cohort of homeless adults with a mental illness and a general cohort of adults with a history of homelessness.

## Methods

This study used data from two prospective studies: the At Home/Chez Soi study and the Health and Housing in Transition study. Survey data were linked with administrative healthcare records in Ontario (accessed at ICES, formerly the Institute for Clinical Evaluative Sciences).

### Data sources

The At Home/Chez Soi study, referred to as the “cohort with a mental illness”, was a randomized controlled trial of Housing First in five Canadian cities: Toronto, Moncton, Montreal, Winnipeg, and Vancouver. At enrolment (2009 to 2011), the participants were at least 18 years old, diagnosed with a mental disorder, and absolutely homeless (i.e., no fixed place to stay for at least the past 7 nights with little likelihood of finding a place in the upcoming month) *or* precariously housed (i.e., housed in single room occupancy, rooming house, or hotel/motel as a primary residence and a history of two or more episodes of being absolutely homeless in the past year or one episode of absolute homelessness lasting at least 4 weeks in the past year). Following enrolment, participants were randomized to receive the Housing First intervention, which included rent supplements to facilitate rapid access to housing with individualized community supports, or treatment as usual. Follow up interviews on health and housing outcomes were conducted every 6 months over a 2-year follow up period [14].

The Health and Housing in Transition study, referred to as the “general homeless cohort”, was a longitudinal cohort study conducted in three Canadian cities: Toronto, Ottawa, and Vancouver. At enrolment (2009), participants were at least 18 years old and either homeless (i.e., living in a shelter, public place, vehicle, abandoned building, or someone else’s place) *or* vulnerably housed *(*i.e.*,* living in their own room, apartment, or place, having been homeless in the past 12 months and/or having two or more moves in the past 12 months). Follow up interviews were completed every 12 months over a 4-year period [15]. Participants provided informed consent for participation in both studies.

Administrative healthcare data for Ontario residents is stored at ICES, a non-for-profit organization that functions as a repository of provincial health records. Under the Ontario Health Insurance Plan (OHIP), a single-payer health system, all Ontario residents are eligible for healthcare coverage. The services covered by OHIP include physician and in-hospital services for the general population and also medication prescriptions for people over 65 years old or those enrolled in social assistance or disability pension programs. Most participants are eligible for these support programs and their prescription costs would therefore be captured in these data (e.g., at least 75% of the participants had prescription costs).

Participants of the At Home/Chez Soi or Health and Housing in Transition studies were eligible for inclusion if they were enrolled in the Ontario study sites and provided informed consented to linkage with administrative healthcare data at ICES. Personal identifiers such as Ontario health card number, name, date of birth, gender, and postal code were used to assign a unique ICES key number (IKN) for linkage across internal and external datasets. Individuals were excluded if they did not consent to data linkage or their personal identifiers could not be matched to a record at ICES.

### Independent variables

Predisposing, enabling, and need factors were identified using the Behavioral Model for Vulnerable Populations [16]. *Predisposing Factors* include age, gender, marital status, racial identity, education, employment, housing history, mental illness, substance use, criminal behaviour, and victimization. *Enabling Factors* include personal and community resources, such as regular source of care, perceived barrier to care, and an indicator of not enough food to eat. *Need factors* include perceived health status and observed health conditions. Complete variable descriptions are previously provided [17].

### Outcome data

Total healthcare costs were estimated using a validated individual-level costing algorithm for all health services covered by OHIP [18]. This algorithm calculated total costs by combining the frequency and intensity of resource utilization with a weighted per unit cost. Individual-level total costs were then classified as being within the top 5%, top 6–10%, top 11–50%, or bottom 50% of healthcare users based on provincial cut-points established from a representative sample of Ontario residents enrolled in the Canadian Community Health Survey [8].

Service-specific healthcare costs were also estimated for psychiatric and non-psychiatric inpatient costs, emergency department costs, outpatient hospital costs including dialysis and cancer clinic services, OHIP physician costs, prescription medication costs, and other costs. The data sources for these encounters were the Ontario Mental Health Reporting System (OMHRS) and the Discharge Abstract Database (CIHI-DAD) for inpatient services, the National Ambulatory Care Reporting System (NACRS) for emergency and outpatient hospital services, OHIP for other physician visits, and the Ontario Drug Benefit (ODB) for prescription medications. Other costs include the remaining services covered by OHIP but not captured in the aforementioned categories. Details on these services are previously described [17]. These datasets were linked using unique encoded identifiers and analyzed at ICES.

### Statistical analysis

All analyses were conducted separately for the cohort with a mental illness and the general homeless cohort. Predisposing, enabling and need characteristics at baseline were reported for the total samples and for participants whose total costs were within the top 5% of users. The reported *p*-values (α = 0.05) were calculated using chi-squared tests for binary variables and analysis of variance or Kruskal-Wallis tests for continuous variables to compare the characteristics of the top 5% and bottom 95% of users.

The proportion of total healthcare costs attributed to each service were described across the four gradient categories (top 5%, top 6–10%, top 11–50%, and bottom 50%). Multinomial logistic regression was used to identify the predisposing, enabling, and need factors associated with higher levels of healthcare expenditure, using ‘bottom 0-50% of healthcare users’ as the reference group [19]. To depict the real-life circumstances where individual characteristics cannot be isolated, the main analyses were unadjusted [20, 21]. Since this analysis does not attempt to draw causal comparisons, adjustment could needlessly distort the observed associations within the cohort. Instead, by reporting unadjusted associations as the main analysis, it becomes clear which factors are associated with higher use of health services to inform the development of tailored interventions. Recognizing that future research may examine a causal relationship between exposures and healthcare costs, we also report the age-adjusted and fully adjusted models in the supplemental file for comparison. Another consideration is the potential impact of the Housing First intervention on the findings within the cohort with a mental illness (At Home/Chez Soi study). A supplementary analysis included an indicator variable for the intervention within each model to assess whether the associations changed following this adjustment.

Missing data was reported as a characteristic and modelled as an exposure for the unadjusted analyses. For the supplementary fully adjusted models, multiple imputation and bootstrapped modelling techniques were applied to estimate the confidence intervals [22]. Multiple imputation was conducted using the mi, fcs command in SAS version 9.4, with the 100 imputed datasets combined using the mianalyze command [23]. Analyses were performed using SAS version 9.4 [23].

This study was conducted in accordance with the Declaration of Helsinki and approved by Research Ethics Boards at St. Michaels Hospital and the University of Toronto.

## Results

The linkage rates were 91% for the cohort with a mental illness (525 of 575 participants) and 85% for the general homeless cohort (677 of 796 participants). Among the 575 people enrolled in the Toronto site of the At Home/Chez Soi study, 8 did not consent to data linkage and 42 could not be linked with administrative data due to invalid ICES key number or death prior to index date. Of the 796 participants enrolled in the Toronto or Ottawa site of the Health and Housing in Transition study, 11 did not consent to linkage, 94 did not have a valid ICES key number, and 14 were deemed ineligible due to OHIP ineligibility, missing data, or death. For the 22 duplicate records, the At Home/Chez Soi index date was retained. Full inclusion criteria are described in Supplemental Fig. S1 and a comparison of the included and excluded sample characteristics are reported in Supplemental Table S1.

Table 1 describes the sample characteristics. For the cohort with a mental illness, people in the top 5% of users were more likely to be single, high school educated, absolutely homeless at enrolment, admitted to hospital in the past 12 months, and criminalized or victimized in the past 6 months compared to the bottom 95% of users. They were also less likely to identify as Black or other racialized groups, report problematic alcohol or drug use, smoke daily, and perceive a barrier to care. For the general homeless cohort, the top 5% of users were more likely to be female, admitted to hospital in the past 12 months, criminalized or victimized in the past 12 months, diagnosed with a psychotic disorder, and to report problematic alcohol or drug use, have a regular medical doctor, or perceive their general health as poor.

**Table 1:** Sample characteristics for the cohort with a mental illness and the general homeless cohort ^a^

Characteristics	Cohort with a mental illness			General homeless cohort			Missing data
	Total Sample (*n* = 525)	Top 5% (*n* = 160)	*p*-value ^b^	Total Sample (*n* = 655)	Top 5% (*n* = 108)	*p*-value ^b^	%
**Predisposing Factors**							
**Age**							–
Mean ± SE	39.9 ± 11.8	40.0 ± 12.4	0.939	42.9 ± 10.5	44.1 ± 9.7	0.220	
Median (IQR)	41 (30–48)	41 (30–48)	0.871	44 (36–50)	45 (38–50)	0.171	
**Age Group**							–
18–34	34.9%	34.4%	0.815	21.8%	16.7%	0.351	
35–49	44.4%	46.3%		51.3%	53.7%		
50+	20.8%	19.4%		26.9%	29.6%		
**Gender**							–
Female	30.1%	32.5%	0.426	30.7%	38.9%	0.043	
Male	69.9%	67.5%		69.3%	61.1%		
**Marital status**							2–3%
Single, never married	69.5%	73.5%	0.20	60.6%	58.3%	0.474	
Widowed, separated, divorced	27.5%	22.4%		28.0%	32.4%		
Partnered, married	3.0%	4.1%		11.3%	9.3%		
**Race**							1–2%
Black	32.4%	28.8%	0.133	12.0%	4.7%	< 0.001	
Other racialized groups	30.9%	28.1%		23.2%	14.4%		
White	36.7%	43.1%		64.8%	80.9%		
**Place of birth**							1–2%
Canada	55.0%	58.7%	0.288	81.8%	89.8%	0.018	
**Education**							2–3%
Graduated high school	51.3%	56.4%	0.139	56.9%	50.9%	0.17	
**Employment Status**							2–3%
Currently employed	4.2%	< 3.8%	0.555	10.4%	< 5.6%	0.032	
**Housing status** ^**c**^							–
Precariously or vulnerably housed	7.4%	4.4%	0.08	50.1%	51.9%	0.686	
Homeless	92.6%	95.6%		49.9%	48.1%		
**Years spent homeless**							4–5%
Mean ± SE	5.21 ± 6.10	4.9 ± 6.0	0.509	5.32 ± 6.22	6.4 ± 6.9	0.049	
Median (IQR)	3 (1–7)	3 (1–6)	0.329	3 (1–7)	4 (1–10)	0.097	
≥ 2 years spent homeless	61.7%	60.6%	0.734	58.6%	61.1%	0.566	
**Criminal behaviour** *(Past 6 to 12 months)*	39.2%	40.7%	0.663	37.1%	42.1%	0.242	1–2%
**Victimization** *(Past 6 to 12 months)*	35.1%	39.2%	0.21	37.2%	43.9%	0.116	2–3%
**Diagnosed mental illness**							–
Psychotic disorder	43.0%	47.5%	0.173	13.3%	29.6%	< 0.001	
Other disorder	57.0%	52.5%		25.6%	31.5%		
No disorder	–	–		61.1%	38.9%		
**Problematic alcohol use** *(Past 12 months)*	45.1%	41.3%	0.235	20.2%	27.8%	0.031	–
**Problematic drug use** *(Past 12 months)*	50.7%	43.1%	0.022	35.9%	52.8%	< 0.001	–
**Smoking status**							2–3%
Current, daily smoker	65.1%	59.1%	0.066	75.5%	76.6%	0.757	
**Enabling Factors**							
**Regular source of care**							
Yes	66.7%	65.3%	0.664	59.2%	76.9%	< 0.001	1–2%
**Perceived barrier to care** *(Past 6 to 12 months)*	40.2%	36.0%	0.211	35.2%	36.8%	0.703	2–3%
**Food insecurity** *(not enough food)*	51.8%	49.0%	0.416	32.2%	34.3%	0.609	1–2%
**Acute mental health care** *(past 12 months)*			< 0.001			< 0.001	–
Hospital admission or emergency department visit	52.8%	77.5%		20.2%	50.0%		
**Acute non-mental health care** *(past 12 months)*			< 0.001			< 0.001	–
Hospital admission or emergency department visit	62.1%	77.5%		47.0%	63.9%		
**Need Factors**							
**Perceived general health**							1–2%
Poor	19.2%	22.7%	0.209	13.1%	25.0%	< 0.001	
Fair	31.7%	26.7%		28.9%	30.6%		
Good, very good, excellent	49.1%	50.7%		58.0%	44.4%		
**Chronic conditions** *(administrative data)*							–
Mean ± SE	0.59 ± 0.84	0.7 ± 0.9	0.005	0.57 ± 0.85	1.0 ± 1.1	< 0.001	
Median (IQR)	0 (0–1)	0 (0–1)	0.008	0 (0–1)	1 (0–2)	< 0.001	
**Chronic conditions** *(survey data)*							1–2%
Mean ± SE	1.01 ± 1.28	1.1 ± 1.5	0.191	1.06 ± 1.25	1.6 ± 1.5	< 0.001	
Median (IQR)	1 (0–1)	1 (0–2)	0.781	1 (0–2)	1 (0–3)	< 0.001	
**Any Missing Data** ^d^	15.4%	21.9%	0.004	7.9%	6.5%	0.54	

^a^Cost gradient cut-offs were applied based on the general Ontario population thresholds: Rosella et al. High-cost healthcare users in Ontario, Canada: demographic, socio-economic, and health status characteristics. *BMC Health Serv Res, 2014; 14*, 532.

^b^Chi-square tests, analysis of variance, and Kruskal Wallace tests were used to compare the top 5% of consumers to the bottom 95% of consumers (α = 0.05).

^c^Small cell sizes of < 6 were reported as the equivalent percent.

^d^Any missing data does not include chronic conditions as administrative data was available

Missing data was also reported in Table 1. The percentage of participants with at least one missing data point was higher in the cohort with a mental illness than the general homeless cohort (15% versus 8%). The predisposing factor with the most missing data was lifetime duration of homelessness at approximately 4–5%. In comparison, the factors that were collected as part of the study enrolment criteria (e.g., housing status) or supplemented with administrative healthcare records (e.g., diagnosed chronic conditions) had the least amount of missing data at 0%.

**Fig. 1:**
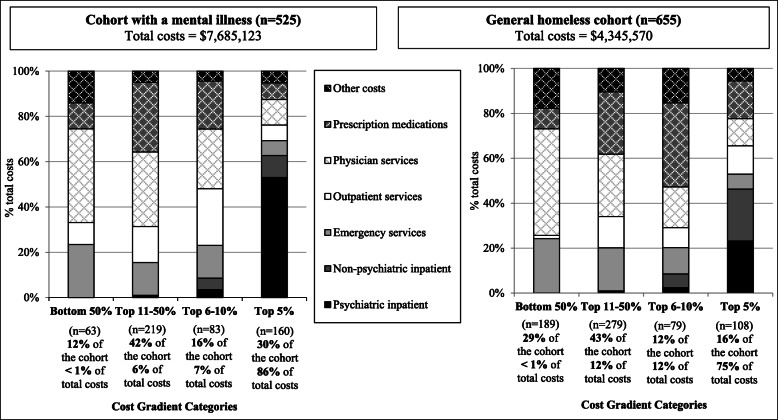
Healthcare cost distribution among the cohort with a mental illness and the general homeless cohort

Figure 1 illustrates the distribution of total and service-specific healthcare costs across the cost gradient categories (top 5%, top 6–10%, top 11–50%, bottom 50%). For the cohort with a mental illness, 12% of the sample were in the bottom 50% of users and incurred less than 1% of total costs, while 30% were in the top 5% of users and incurred 86% of the total costs. The proportion of total costs atributed to inpatient costs increased from 0% for the bottom 50% of users to 63% for the top 5% of users. Concurrently, the proportion of total costs attributed to physician services decreased from 41 to 11% across the gradient categories. Similar patterns were observed for the general homeless cohort, where 29% of the sample were in the bottom half of users and incurred less than 1% of total costs and 16% of the sample were in the top 5% of users and incurred 75% of total costs. The proportion of total costs attributed to psychiatric and non-psychiatric inpatient services increased from 0 to 46%, while the proportion attributed to physician services decreased from 48 to 12% across the cost gradient categories.

Table 2 reports the average healthcare costs per person in each cohort across the gradient categories. From the bottom 50% to the top 5% of users, the mean (and median) total costs increased from $158 to $41,425 (and $21 to $3545) for the cohort with a mental illness, and $115 to $30,269 (and $20 to $2271) for the general homeless cohort. Inpatient costs were a main contributor to this increase. In the cohort with a mental illness, mean inpatient costs increased from $0 to $21,951 for psychiatric admissions and $0 to $4010 for non-psychiatric admissions. In the general homeless cohort, similar mean increases in costs were observed for psychiatric ($0 to $7012) and non-psychiatric ($0 to $6990) admissions. It is notable that all of the service-specific costs increased across the gradient categories within both cohorts.

**Table 2:** Average healthcare costs for the cohort with a mental illness and the general homeless cohort

Survey		Cohort with a mental illness (***n*** = 525)				General homeless cohort (***n*** = 655)			
Cost Categories		Bottom 0–50% (*n* = 63)	Top 11–50% (*n* = 219)	Top 6–10% (*n* = 83)	Top 5% (*n* = 160)	Bottom 0–50% (*n* = 189)	Top 11–50% (*n* = 279)	Top 6–10% (*n* = 79)	Top 5% (*n* = 108)
Total costs	Mean ± SE	$158 ± $17	$2222 ± $83	$6754 ± $153	$41,425 ± $3674	$115 ± $9	$1872 ± $68	$6743 ± $151	$30,269 ± $3699
	Median (IQR)	$170 ($0–$274)	$1969 ($1154–$3214)	$6498 ($5620–$7848)	$24,265 ($13,859–$43,959)	$78 ($0–$206)	$1564 ($857–$2765)	$6575 ($5591–$7879)	$17,755 ($12,511–$31,939)
Psychiatric inpatient costs	Mean ± SE	$0	$21 ± $12)	$240 ± $111	$21,951 ± $3082	$0	$15 ± $11	$157 ± $73)	$7012 ± $2011
	Median (IQR)	$0	$0	$0	$5809 (0-$27,543)	$0	$0	$0	$0 (0-$6276)
Non-psychiatric inpatient costs	Mean ± SE	$0	$0	$335 ± $119	$4010 ± $1262	$0	$2 ± $2	$416 ± $131	$6990 ± $2380
	Median (IQR)	$0	$0	$0	$0 (0-$1007)	$0	$0	$0	$0 (0-$6365)
Emergency department costs	Mean ± SE	$37 ± $10	$322 ± 37	$980 ± 129	$2758 ± 490	$28 ± 5	$359 ± 20	$791 ± 122	$2019 ± 228
	Median (IQR)	$0	$101 ($0–$433)	$563 ($0–$509)	$1355 ($583–$2833)	$0	$166 ($0–$490)	$315 ($0–$1322)	$1332 ($505–$2660)
Hospital outpatient costs	Mean ± SE	$15 ± $9	$355 ± 42	$1690 ± 196	$2825 ± 301	$2 ± 2	$261 ± 29	$597 ± 96	$3818 ± 1259
	Median (IQR)	$0	$0 ($0–$323)	$966 ($323–$2899)	$1613 ($645–$3868)	$0	$0 ($0–$304)	$304 ($0–$666)	$1264 ($627–$2811)
Physician service costs	Mean ± SE	$65 ± $11	$731 ± $43	$1782 ± $131	$4685 ± $346	$54 ± $6	$520 ± $27	$1227 ± $79	$3656 ± $450
	Median (IQR)	$21 ($0–$113)	$546 ($284–$961)	$1517 ($959–$2299)	$3545 ($2147–$5602)	$20 ($0–$84)	$376 ($196–$722)	$1181 ($634–$1655)	$2271 ($1452–$3513)
Prescription medication costs	Mean ± SE	$18 ± 6	$681 ± 51	$1431 ± 168	$3029 ± 479	$10 ± 2	$519 ± 44	$2522 ± 212	$5085 ± 561
	Median (IQR)	$0 ($0–$12)	$464 ($103–$992)	$953 ($209–$2205)	$1388 ($500–$3306)	$0	$160 ($21–$821)	$2219 ($882–$3664)	$3502 ($400–$8382)
Other service costs	Mean ± SE	$22 ± 6	$112 ± 15	$296 ± 51	$2167 ± 816	$20 ± 3	$195 ± 22	$1033 ± 140	$1690 ± 357
	Median (IQR)	$0 ($0–$14)	$45 ($0–$147)	$96 ($1–$332)	$95 ($1–$536)	$0 ($0–$19)	$66 ($0–$183)	$290 ($127–$1610)	$218 ($39–$1773)

Table 3 reports the unadjusted multinomial odds ratio estimates for factors associated with membership in each cost gradient category, using the bottom 50% of users as the reference. For both cohorts, criminal behaviour, victimization, and reporting a regular source of care were associated with higher odds of being in any of the higher cost categories. Past year acute mental and non-mental health care and ≥ 2 chronic conditions were associated with higher odds of high cost service use, with odds ratios increasing across the gradient categories. Single marital status and current employment were associated with lower odds of being in a higher cost use category. Black participants also had lower odds of being a higher cost user, with stronger odds ratios across the gradient categories.

**Table 3:** Unadjusted odds ratios for the predisposing, enabling, and need factors associated with higher cost use

Study	Odds Ratio (95%CI)					
	Cohort with a mental illness (***n*** = 525)			General homeless cohort (***n*** = 655)		
Cost category	Top 11–50% (*n* = 219)	Top 6–10% (*n* = 83)	Top 5% (*n* = 160)	Top 11–50% (*n* = 279)	Top 6–10% (*n* = 79)	Top 5% (*n* = 108)
**Predisposing Factors**						
**Age**						
Per 1-year increase	1.00 (0.98, 1.03)	1.01 (0.98, 1.04)	1.01 (0.98, 1.03)	1.00 (0.98, 1.02)	1.00 (0.98, 1.03)	1.01 (0.99, 1.04)
**Age Group** *18 to 34 years (reference)*						
35 to 49	1.87 (0.98, 3.57)	2.18 (1.02, 4.68)	1.88 (0.96, 3.69)	0.98 (0.61, 1.55)	1.23 (0.62, 2.42)	1.47 (0.78, 2.79)
50+	1.10 (0.53, 2.27)	1.42 (0.60, 3.37)	1.05 (0.49, 2.26)	0.90 (0.53, 1.52)	1.03 (0.48, 2.24)	1.47 (0.73, 2.97)
**Gender** ^a^						
Female	1.68 (0.87, 3.24)	1.18 (0.55, 2.57)	1.68 (0.85, 3.32)	1.83 (1.19, 2.82)	2.35 (1.33, 4.18)	2.45 (1.45, 4.13)
**Marital Status**						
Single, never married	0.71 (0.37, 1.36)	0.55 (0.26, 1.14)	0.92 (0.46, 1.84)	0.66 (0.45, 0.97)	0.75 (0.43, 1.29)	0.69 (0.42, 1.12)
**Race**						
Black	0.68 (0.33, 1.40)	0.46 (0.20, 1.04)	0.43 (0.21, 0.92)	0.97 (0.56, 1.69)	0.55 (0.23, 1.35)	0.30 (0.11, 0.81)
Other racialized groups	0.60 (0.29, 1.23)	0.32 (0.14, 0.75)	0.39 (0.19, 0.82)	1.02 (0.65, 1.62)	0.70 (0.35, 1.39)	1.09 (0.63, 1.92)
**Place of Birth**						
Outside Canada	1.26 (0.71, 2.23)	0.71 (0.36, 1.39)	0.86 (0.47, 1.57)	1.08 (0.68, 1.73)	0.99 (0.51, 1.93)	0.48 (0.23, 0.99)
**Education**						
Graduated high school	0.78 (0.44, 1.39)	0.92 (0.47, 1.79)	1.13 (0.62, 2.06)	0.75 (0.52, 1.10)	0.75 (0.44, 1.28)	0.62 (0.38, 1.00)
**Employment**						
Currently employed	0.31 (0.10, 0.95)	0.34 (0.08, 1.43)	0.31 (0.09, 1.07)	0.56 (0.32, 0.99)	0.62 (0.27, 1.42)	0.27 (0.10, 0.71)
**Housing Status**						
Homeless	0.67 (0.22, 2.05)	0.64 (0.18, 2.21)	1.48 (0.42, 5.25)	0.71 (0.49, 1.03)	0.75 (0.43, 1.29)	0.73 (0.45, 1.17)
**Duration of homelessness**						
Per 1-year increase	0.98 (0.94, 1.03)	1.00 (0.95, 1.05)	0.98 (0.93, 1.03)	0.99 (0.96, 1.03)	0.98 (0.94, 1.03)	1.03 (0.99, 1.06)
≥ 2 years spent homeless	1.02 (0.57, 1.82)	0.75 (0.38, 1.47)	0.89 (0.48, 1.62)	1.02 (0.70, 1.49)	0.90 (0.53, 1.53)	1.13 (0.70, 1.83)
**Criminal behavior** *(past 6 to 12 months)*	1.88 (0.98, 3.58)	2.86 (1.38, 5.91)	2.06 (1.05, 4.01)	1.45 (0.98, 2.15)	1.56 (0.91, 2.70)	1.67 (1.02, 2.73)
**Victimization** *(past 6 to 12 months)*	2.01 (1.02, 3.94)	2.02 (0.94, 4.33)	2.33 (1.16, 4.68)	1.71 (1.15, 2.54)	1.64 (0.95, 2.86)	1.99 (1.21, 3.28)
**Diagnosed of mental illness**						
Psychotic disorder	0.81 (0.41, 1.60)	1.16 (0.69, 1.94)	1.37 (0.80, 2.35)	0.92 (0.33, 2.56)	1.71 (0.57, 5.20)	2.51 (0.87, 7.21)
Other disorder *(reference)*	–	–	–	–		–
No disorder	–	–	–	0.17 (0.09, 0.30)	0.11 (0.06, 0.23)	0.12 (0.06, 0.23)
**Substance use**						
Problematic alcohol use	1.32 (0.74, 2.34)	2.34 (1.20, 4.58)	1.14 (0.63, 2.07)	1.64 (0.99, 2.73)	1.85 (0.95, 3.61)	2.41 (1.34, 4.35)
Problematic drug use	1.24 (0.71, 2.17)	1.75 (0.90, 3.40)	0.83 (0.46, 1.50)	3.10 (1.96, 4.91)	7.01 (3.88, 12.68)	5.92 (3.44, 10.20)
Current daily smoker	0.97 (0.52, 1.79)	0.94 (0.46, 1.93)	0.67 (0.35, 1.26)	0.97 (0.63, 1.49)	1.01 (0.54, 1.86)	1.06 (0.61, 1.86)
Enabling Factors						
**Regular source of care**						
Yes	2.85 (1.58, 5.13)	4.05 (1.97, 8.35)	2.30 (1.25, 4.24)	2.08 (1.43, 3.02)	4.11 (2.29, 7.36)	4.62 (2.71, 7.87)
**Perceived barrier to care** *(past 6 to 12 months)*	1.18 (0.65, 2.12)	1.26 (0.64, 2.48)	0.90 (0.49, 1.68)	1.87 (1.24, 2.81)	2.57 (1.48, 4.47)	1.75 (1.04, 2.92)
**Food insecurity** *(not enough food)*	1.37 (0.77, 2.43)	1.32 (0.68, 2.58)	1.10 (0.60, 2.00)	0.87 (0.58, 1.30)	1.64 (0.95, 2.82)	1.13 (0.68, 1.87)
**Acute mental health care** *(past 12 months)*	4.27 (2.00, 9.08)	10.60 (4.60, 24.45)	20.67 (9.31, 45.87)	4.37 (2.09, 9.12)	6.33 (2.72, 14.75)	20.00 (9.27, 43.13)
**Acute non-mental health care** *(past 12 months)*	2.26 (1.26, 4.04)	4.86 (2.40, 9.86)	6.42 (3.39, 12.14)	2.87 (1.92, 4.30)	5.99 (3.38, 10.61)	5.20 (3.12, 8.67)
**Need Factors**						
**Perceived general health**						
Fair	1.04 (0.55, 1.96)	0.77 (0.36, 1.63)	0.73 (0.37, 1.43)	1.41 (0.91, 2.17)	2.89 (1.60, 5.20)	2.05 (1.17, 3.58)
Poor	1.10 (0.49, 2.48)	1.01 (0.40, 2.56)	1.30 (0.57, 2.96)	1.85 (0.95, 3.60)	3.30 (1.40, 7.79)	5.26 (2.55, 10.87)
**Diagnosed conditions** *(administrative records)*						
1	1.62 (0.80, 3.27)	2.05 (0.91, 4.60)	2.24 (1.08, 4.65)	3.10 (1.89, 5.10)	5.67 (2.96, 10.87)	3.66 (1.94, 6.92)
2+	2.04 (0.67, 6.19)	4.27 (1.33, 13.77)	4.44 (1.48, 13.36)	3.39 (1.50, 7.63)	9.45 (3.74, 23.91)	14.84 (6.43, 34.21)
**Self-reported conditions** *(survey data)*						
1	1.58 (0.81, 3.09)	1.27 (0.57, 2.86)	1.21 (0.59, 2.46)	1.93 (1.25, 2.97)	2.28 (1.18, 4.39)	1.66 (0.89, 3.09)
2+	1.50 (0.70, 3.23)	2.38 (1.02, 5.57)	1.67 (0.76, 3.67)	3.28 (1.93, 5.57)	6.14 (3.07, 12.25)	7.34 (3.95, 13.66)
**Missing Data**	0.81 (0.36, 1.83)	0.73 (0.27, 1.96)	1.68 (0.76, 3.74)	0.69 (0.36, 1.33)	0.74 (0.28, 1.92)	0.62 (0.25, 1.53)

For the cohort with a mental illness, the odds of being a higher cost user were approximately 2 times higher for people 35 to 49 years old across all gradient categories. For the general homeless cohort, the odds of being in a high-cost category were higher for females and people with a perceived barrier to care, problematic alcohol or drug use, regular source of care and poor perceived general health, while high school education was associated with lower odds of being a higher cost user.

Supplemental Tables S2 and S3 report the age-adjusted and fully adjusted multinomial logistic regression model estimates. The unadjusted and age-adjusted models were similar; however, certain associations were attenuated in the fully adjusted models. For instance, the associations for criminal behaviour and victimization were attenuated in the cohort with a mental illness, and nearly reached the null value within the general homeless cohort. Other factors remained associated with higher cost use including regular source of care, and past year acute mental or non-mental health care. Within the general homeless cohort, specifically, female gender, psychotic disorder, problematic drug use, poor perceived health, and diagnosed chronic conditions were associated with higher cost use in the fully adjusted models. Subsequently, the models adjusted for the Housing First intervention in the At Home/Chez Soi study (Supplemental Table S4) were similar to the unadjusted models, which provided no indication that the intervention altered the strength or precision of the estimates.

## Discussion

This study’s findings advance the literature on healthcare costs within the homeless population by leveraging healthcare data from provincial administrative records and applying data linkage with survey data from two large cohort of adults experiencing homelessness. The application of established cost-gradient categories from the general Ontario population enables comparability of results across the two homeless cohorts and with previous findings from the Ontario housed population. For instance, an important finding was the high proportion of participants with healthcare costs in the top 5% of Ontario healthcare users – reaching 30% of the cohort with a mental illness and 16% of the general homeless cohort. The total costs for participants in the top 5% user category were largely attributed to inpatient services for both cohorts; however, mean costs were higher for the cohort with a mental illness than the general homeless cohort ($41,425 versus $30,269). This difference is primarily attributed to higher mean psychiatric inpatient costs within the cohort with a mental illness ($21,951 versus $7012), which accounted for 53% of costs for the cohort with a mental illness and 23% of total costs for the general homeless cohort. In comparison, the average total and service-specific costs for the bottom 50% of users were similar across the cohort with a mental illness and the general homeless cohort. This finding is not exclusive to the homeless population; inpatient care is similarly a large contributor to the healthcare costs consumed by the top 5% in the general population [8].

This study also examined the determinants of higher cost use within the homeless cohorts to understand the characteristics of patients who require the most services and to inform healthcare-driven support services for people experiencing homelessness. Criminal behaviour and victimization were associated with higher cost use for both cohorts, with stronger associations for the cohort with a mental illness. Conversely, the associations for problematic drug use, chronic conditions, perceived barrier to care, and poor perceived general health were stronger among the general homeless cohort. These observed differences in strength of association may be explained by study enrolment criteria, as the cohort with a mental illness was absolutely homeless and had a diagnosable mental disorder at enrolment, while the general homeless cohort did not need to meet these criteria [15, 24].

Some of the factors associated with higher cost use within the two homeless cohorts were similar to previous findings from the general Ontario housed population [8]. For instance, participants who reported a regular source of care had higher odds of being a higher cost user in the homeless cohorts and the housed population. People with a regular source of health care may be more connected with services or have higher morbidity that also requires expensive acute services. Alternatively, some individuals may receive inadequate primary care that contributes to a need for acute services. Female gender and poor perceived health were also associated with higher cost use, while an inverse association was observed for Black participants in the homeless cohorts and housed population [8]. Past research demonstrates that visible minority groups experience discrimination in healthcare settings more often than white patients, which can reduce their willingness to seek care when necessary [25].

There were other associations from the homeless cohorts that differed from the Ontario housed population. For instance, older age was not consistently associated with higher cost use in either homeless cohort; yet age is a strong determinant of higher cost use within the Ontario general population. People experiencing homelessness have a higher prevalence of mental illness and risk of premature mortality than the general population, which can contribute to costly service use at younger ages [9, 26, 27]. Finally, criminal behaviour, victimization, substance use and psychotic disorders were also associated with higher cost use within the homeless cohorts specifically. By understanding the factors that are unique to the homeless population, these findings highlight the types of support services that may be required to provide tailored approaches to address homelessness. For instance, victimization and criminal behaviour were associated with higher cost use, which suggests a need for trauma-informed supports and access to legal services for people experiencing homelessness who present to hospital (as inpatient and emergency services were a large contributor to healthcare costs). The associations for psychotic disorder and substance use suggest a need for mental health and substance rehabilitation supports for people who use the healthcare system while homeless.

### Strengths and limitations

This study’s strengths centre on the application of data linkage to combine individual-level survey data from two cohorts of homeless adults with comprehensive administrative healthcare records in Ontario. Combining these data sources fills the gaps that exist when using survey or administrative data in isolation. For instance, administrative records may lack in-depth personal information on social factors, housing history, and health behaviours, while survey healthcare utilization data are often incomplete or limited by self-report. The comprehensive assessment of healthcare costs in this study was uniquely achievable due to the single-payer healthcare system in Ontario and the use of a validated algorithm to calculate person-level healthcare costs [18]. Further, the use of cost gradient categories that were established from previous work in the general Ontario population, enabled a more direct comparison of the cohort of homeless adults with a mental illness and the general homeless cohort (as did the similar timing of data collection and eligibility criteria for the two studies). This work highlights the diversity of healthcare costs within the homeless population and identifies factors to consider when implementing healthcare-driven housing and support interventions for homeless patients.

There are also some limitations that must be considered when interpreting these findings. First, not all participants could be linked to administrative health records due to lack of consent or insufficient personal identifiers. This was the case for 9% of the cohort with a mental illness (*n* = 50) and 15% of the general homeless cohort (*n* = 119). Compared with the participants who could not be linked, the included sample was more likely to report a usual source of healthcare for both the cohort with a mental illness (67% vs 51%) and the general homeless cohort (59% vs 49%). For the general homeless cohort specifically, the included sample was also older (43 years vs 39 years) and less likely to perceive a barrier to care (35% vs 44%). This may suggest that the included sample was more connected to services than the excluded sample. Second, not all healthcare encounters could be linked to an individual due to missing information on personal identifiers at encounter. For instance, each year it is estimated that 1–2% of hospitalizations and ED visits in the province are not attached to an individual due to missing health card number or other identifiers [28–30]. The costing algorithm uses healthcare utilization data to estimate costs, so it is possible that total costs were underestimated for some participants. Further, this analysis takes a payer perspective, which means the data only capture costs for services that are covered by OHIP and documented in administrative healthcare records. Costs for other services, such as healthcare provided at community health centers, non-hospital dental services, physiotherapy, ambulance co-payments, and prescription medications for people under 65 years old who do not qualify for social assistance or disability pension programs are not included. Therefore, from a patient perspective, the total costs would likely be underestimated. Third, not all relevant factors were assessed during the interview, including accessibility of health and community resources, and veteran status. Fourth, characteristics such as being married or currently employed were less frequently reported, which contributed to imprecision of the estimates and required that certain categories be combined (e.g., married/partnered and widowed/separated/divorced).

## Conclusion

This study combines individual-level survey data with provincial administrative healthcare records to offer a unique view of healthcare costs among people experiencing homelessness. The distribution of healthcare costs provides information about how services are being used by people experiencing homelessness, which can inform future allocation of resources. Understanding the factors associated with higher cost use can further inform the development of targeted interventions that leverage the healthcare system as a point of contact to intervene against homelessness. Future work should examine flexible and tailored housing and support interventions within the healthcare system that can be modified to meet individual needs. It is imperative to recognize that racialized groups may benefit less from healthcare-driven strategies to address homelessness. Therefore, other non-healthcare strategies must be considered to ensure equitable access to housing and support services.

## Supplementary Information

**Additional file 1: Supplemental Figure S1.** Flow chart for the inclusion and exclusion criteria for participants in the cohort with a mental illness (At Home / Chez Soi study) and the general cohort of adults with a history of homelessness (Health and Housing in Transition study). **Supplemental Table S1.** Comparison of the included and excluded samples for the cohort with a mental illness (At Home/Chez Soi study) and the general homeless cohort (Health and Housing in Transition study). **Supplemental Table S2.** Age adjusted, and fully adjusted and imputed odds ratio estimates for the associations between predisposing, enabling, and need factors and healthcare cost categories for the cohort with a mental illness (At Home/Chez Soi study). **Supplemental Table S3.** Fully adjusted and imputed odds ratio estimates for the associations between predisposing, enabling, and need factors and higher cost healthcare use for the general homeless cohort (Health and Housing in Transition study). **Supplemental Table S4.** Multinomial odds ratio estimates for the associations between predisposing, enabling, and need factors and healthcare expenditure categories for the At Home / Chez Soi participants, adjusting for the Housing First intervention (*n*=525).

## Data Availability

The datasets generated and analyzed during the current study are not publicly available due to data sharing agreements and privacy policies that prohibit ICES from sharing the dataset publicly. The datasets supporting the conclusions of this article are held securely in coded form at ICES. SWH can be contacted for any data requests. Upon reasonable request, confidential access may be granted to those who meet pre-specified criteria.
