# Supplementary material for: Factors associated with higher healthcare costs in a cohort of homeless adults with a mental illness and a general cohort of adults with a history of homelessness
Source: BMC Health Serv Res. 2021 Jun 6;21:555. doi: 10.1186/s12913-021-06562-6 (PMC8180071; doi:10.1186/s12913-021-06562-6)
Supplement: Supplementary file 1 — Additional file 1: Supplemental Figure S1. Flow chart for the inclusion and exclusion criteria for participants in the cohort with a mental illness (At Home / Chez Soi study) and the general cohort of adults with a history of homelessness (Health and Housing in Transition study). Supplemental Table S1. Comparison of the included and excluded samples for the cohort with a mental illness (At Home/Chez Soi study) and the general homeless cohort (Health and Housing in Transition study). Supplemental Table S2. Age adjusted, and fully adjusted and imputed odds ratio estimates for the associations between predisposing, enabling, and need factors and healthcare cost categories for the cohort with a mental illness (At Home/Chez Soi study). Supplemental Table S3. Fully adjusted and imputed odds ratio estimates for the associations between predisposing, enabling, and need factors and higher cost healthcare use for the general homeless cohort (Health and Housing in Transition study). Supplemental Table S4. Multinomial odds ratio estimates for the associations between predisposing, enabling, and need factors and healthcare expenditure categories for the At Home / Chez Soi participants, adjusting for the Housing First intervention (n=525). [file 12913_2021_6562_MOESM1_ESM.docx]

***Supplemental Files***

**Title:** Factors associated with higher healthcare costs in a cohort of homeless adults with a mental illness and a general cohort of adults with a history of homelessness

**Kathryn Wiens, MSc,** Dalla Lana School of Public Health, University of Toronto, 155 College St. Toronto Ontario, M5T 1P8 (Email address: kathryn.wiens@mail.utoronto.ca)

**Laura C Rosella, PhD** Dalla Lana School of Public Health, University of Toronto, 155 College St. Toronto Ontario, M5T 1P8 (Email address: laura.rosella@utoronto.ca)

**Paul Kurdyak, MD, PhD** Centre for Addiction and Mental Health, 33 Russel St. Toronto, Ontario, M5S 3M1 (Email address: Paul.Kurdyak@camh.ca)

**Simon Chen, MPH,** ICES, 2075 Bayview Avenue, Toronto Ontario, M4N 3M5 (Email address: simon.chen@ices.on.ca)

**Tim Aubry**, **PhD,** School of Psychology & Centre for Research on Educational and Community Services, University of Ottawa, 136 Jean-Jacques-Lussier Private, Ottawa, Ontario, K1N 9A8 (Email address: Tim.Aubry@uottawa.ca)

**Vicky Stergiopoulos**, **MD, MSc,** Centre for Addiction and Mental Health, 33 Russel St. Toronto, Ontario, M5S 3M1 (Email address: Vicky.Stergiopoulos@camh.ca)

**Stephen W Hwang,** **MD, MPH,** MAP Centre for Urban Health Solutions, St. Michaels Hospital, 30 Bond St. Toronto, Ontario, M5B 1X1 (Email address: Stephen.Hwang@unityhealth.to)

**Corresponding author:** Kathryn Wiens (email: kathryn.wiens@mail.utoronto.ca)

***Supplemental Figure S1:*** Flow chart for the inclusion and exclusion criteria for participants in the cohort with a mental illness (At Home / Chez Soi study) and the general cohort of adults with a history of homelessness (Health and Housing in Transition study).


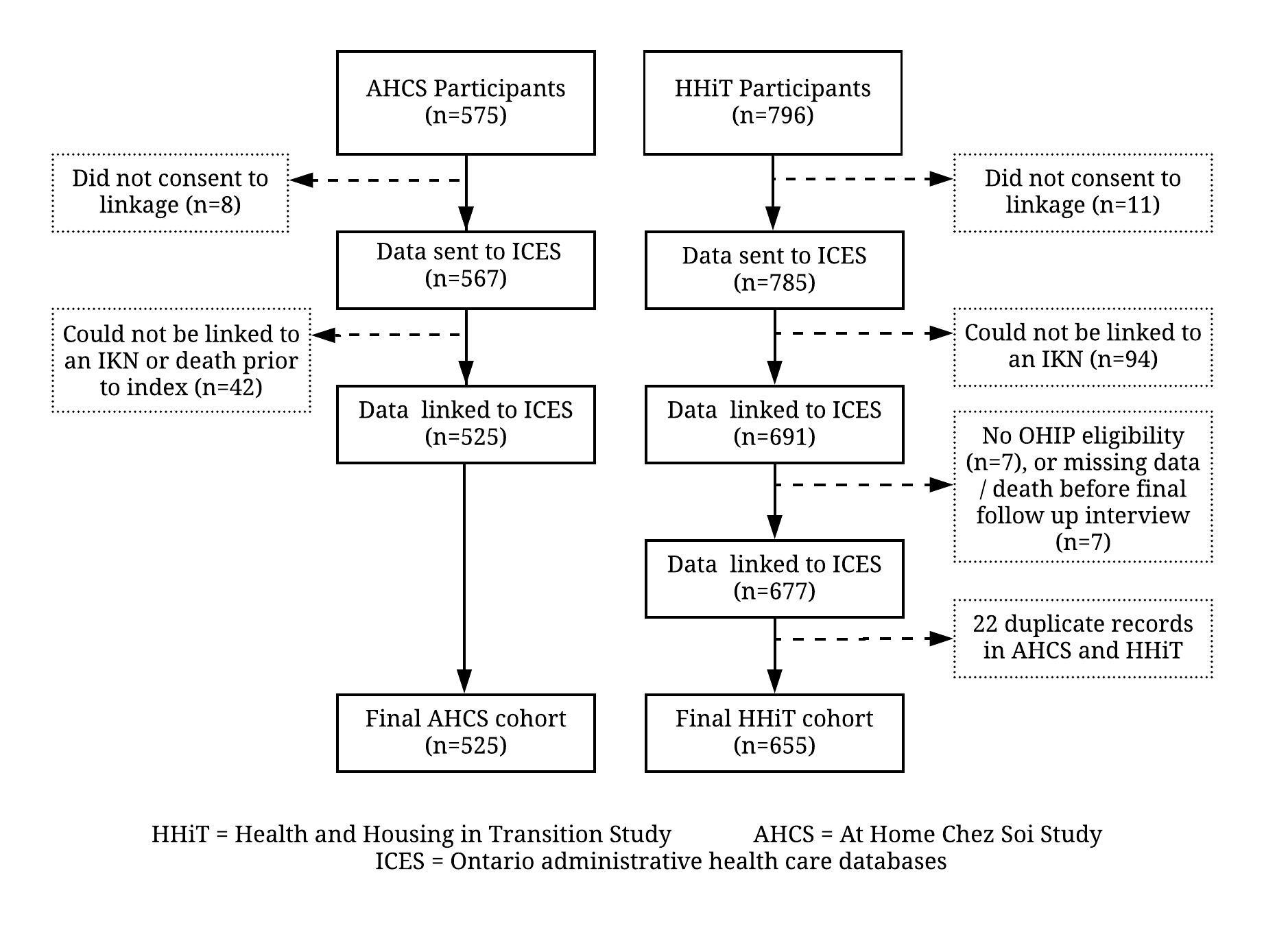


*Note: The 22 individuals enrolled as participants in both studies were retained as participants in the At Home/Chez Soi study.*

***Supplemental Table S1:*** Comparison of the included and excluded samples for the cohort with a mental illness (At Home/Chez Soi study) and the general homeless cohort (Health and Housing in Transition study).

|  | Cohort with mental illness | | | General homeless cohort | | |
| --- | --- | --- | --- | --- | --- | --- |
|  | Included (n=525) | Excluded (n=42) | p-value | Included (n=655) | Excluded (n=108) | p-value |
| **Age**  Mean ± SE | 40.0 ± 11.9 | 38.0 ± 10.8 | 0.314 | 42.9 ± 10.5 | 38.8 ± 10.8 | <0.001 |
| Median (IQR) | 41 (30-48) | 38 (27-48) | 0.322 | 44 (36-50) | 39 (32-46) | <0.001 |
| **Gender** |  |  |  |  |  |  |
| Female | 30.3% | 31.6% | 0.863 | 30.7% | 33.7% | 0.544 |
| Male | 69.7% | 68.4% |  | 69.3% | 66.3% |  |
| **Marital Status**  Single | 69.5% | 70.3% | 0.207 | 60.6% | 59.8% | 0.065 |
| Widowed, Separated, Divorced | 30.5% | 29.7% |  | 28.0% | 21.5% |  |
| Married, Partnered | - | - |  | 11.3% | 18.7% |  |
| **Race**  Black or other racialized groups | 58.3% | 66.7% | 0.288 | 16.2% | 17.0% | 0.850 |
| **Born in Canada**  Yes | 55.0% | 43.2% | 0.164 | 81.8% | 77.6% | 0.299 |
| **Education**  High School | 51.3% | 59.5% | 0.337 | 56.9% | 50.0% | 0.184 |
| **Employment**  Currently employed | - | - |  | 10.4% | 11.2% | 0.798 |
| **Years spent homeless**  Mean ± SE | 5.2 ± 6.1 | 5.8 ± 7.2 | 0.568 | 5.3 ± 6.2 | 4.2 ± 4.9 | 0.07 |
| Median (IQR) | 3 (1-7) | 2 (0-9) | 0.693 | 3 (1-7) | 3 (2-6) | 0.459 |
| **Criminal behaviour**  *(Past 6 to 12 months)* | 39.2% | 45.9% | 0.419 | 37.1% | 38.3% | 0.803 |
| **Victimization**  *(Past 6 to 12 months)* | 35.1% | 29.7% | 0.511 | 37.2% | 43.9% | 0.185 |
| **Diagnosed mental illness** Psychotic disorder | 43.0% | 35.7% | 0.355 | 4.6% | 5.7% | 0.888 |
| Other disorder | 57.0% | 64.3% |  | 44.3% | 43.4% |  |
| No disorder | - | - |  | 51.1% | 50.9% |  |
| **Problematic alcohol use**  *(Past 12 months)* | 41.1% | 31.0% | 0.195 | 15.8% | 16.8% | 0.783 |
| **Problematic drug use**  *(Past 12 months)* | 44.0% | 42.9% | 0.886 | 23.6% | 29.0% | 0.232 |
| **Smoking status**  Current, daily smoker | 65.1% | 73.0% | 0.329 | 75.5% | 78.5% | 0.495 |
| **Regular source of care**  Yes | 66.7% | 51.4% | 0.057 | 59.2% | 49.1% | 0.049 |
| **Perceived barrier to care**  *(Past 6 to 12 months)* | 40.2% | 27.0% | 0.113 | 35.2% | 43.9% | 0.081 |
| **Food insecurity**  *(not enough food)* | 51.8% | 67.6% | 0.063 | 32.2% | 36.4% | 0.381 |
| **Perceived general health**  Poor | 19.2% | 24.3% | 0.701 | 13.1% | 9.3% | 0.246 |
| Fair | 31.7% | 32.4% |  | 28.9% | 24.3% |  |
| Good to Excellent | 49.1% | 43.2% |  | 58.0% | 66.4% |  |
| **Chronic conditions**  *(survey data)*  Mean ± SE | 1.0 ± 1.3 | 1.3 ± 1.4 | 0.239 | 1.1 ± 1.3 | 0.9 ± 1.2 | 0.104 |
| Median (IQR) | 1 (0-1) | 1 (0-2) | 0.197 | 1 (0-2) | 0 (0-1) | 0.056 |

***Supplemental Table S2:*** Age adjusted, and fully adjusted and imputed odds ratio estimates for the associations between predisposing, enabling, and need factors and healthcare cost categories for the cohort with a mental illness (At Home/Chez Soi study).

| Study | Odds Ratio (95%CI) | | | | | |
| --- | --- | --- | --- | --- | --- | --- |
|  | Age-adjusted | | | Fully adjusted | | |
| Cost gradient categories (based on Ontario population) | Top 11-50%  (n=219) | Top 6-10%  (n=83) | Top 5%  (n=160) | Top 11-50%  (n=279) | Top 6-10%  (n=79) | Top 5%  (n=108) |
| Predisposing Factors |  |  |  |  |  |  |
| **Age**  *(per 1 year)* | - | - | - | 1.02 (0.99, 1.06) | 1.03 (0.99, 1.07) | 1.03 (0.99, 1.07) |
| **Gender** ^a^  Female | 1.68 (0.87, 3.25) | 1.20 (0.55, 2.60) | 1.69 (0.86, 3.35) | 1.72 (0.79, 3.76) | 1.20 (0.47, 3.06) | 1.24 (0.52, 2.95) |
| **Marital Status**  Single, never married | 0.71 (0.35, 1.44) | 0.58 (0.26, 1.28) | 1.01 (0.48, 2.12) | 0.92 (0.62, 1.36) | 0.78 (0.49, 1.23) | 1.15 (0.74, 1.78) |
| **Race**  Black | 0.68 (0.33, 1.42) | 0.47 (0.21, 1.09) | 0.43 (0.20, 0.93) | 0.86 (0.54, 1.37) | 0.91 (0.52, 1.60) | 0.74 (0.44, 1.24) |
| Other racialized groups | 0.60 (0.29, 1.24) | 0.33 (0.14, 0.77) | 0.39 (0.19, 0.82) | 0.95 (0.61, 1.48) | 0.75 (0.44, 1.31) | 0.90 (0.54, 1.48) |
| **Place of Birth**  Outside Canada | 1.25 (0.70, 2.22) | 0.68 (0.34, 1.35) | 0.85 (0.46, 1.55) | - | - | - |
| **Education**  Graduated high school | 0.76 (0.43, 1.37) | 0.87 (0.44, 1.71) | 1.11 (0.60, 2.04) | 0.61 (0.30, 1.23) | 0.63 (0.28, 1.46) | 0.82 (0.37, 1.79) |
| **Employment**  Currently employed | 0.31 (0.10, 0.95) | 0.34 (0.08. 1.44) | 0.31 (0.09, 1.07) | 0.31 (0.08, 1.19) | 0.38 (0.07, 2.12) | 0.35 (0.07, 1.73) |
| **Housing Status**  Homeless | 0.67 (0.22, 2.05) | 0.63 (0.18, 2.20) | 1.48 (0.42, 5.23) | 0.48 (0.14, 1.71) | 0.34 (0.08, 1.44) | 0.65 (0.15, 2.88) |
| **Duration of homelessness**  (per 1-year increase) | 0.98 (0.93, 1.02) | 0.99 (0.94, 1.05) | 0.97 (0.93, 1.02) | 0.97 (0.91, 1.02) | 0.98 (0.91, 1.05) | 0.97 (0.91, 1.04) |
| ≥ 2 years spent homeless | 1.01 (0.57, 1.82) | 0.74 (0.38, 1.45) | 0.88 (0.48, 1.61) | - | - | - |
| **Criminal behavior**  *(past 6 to 12 months)* | 1.98 (1.02, 3.83) | 3.23 (1.53, 6.80) | 2.21 (1.11, 4.37) | 1.75 (0.80, 3.81) | 2.16 (0.88, 5.29) | 1.55 (0.66, 3.67) |
| **Victimization**  *(past 6 to 12 months)* | 2.04 (1.04, 4.02) | 2.08 (0.97, 4.49) | 2.39 (1.19, 4.81) | 1.56 (0.72, 3.38) | 1.29 (0.52, 3.17) | 1.86 (0.79, 4.41) |
| **Diagnosed of mental illness**  Psychotic disorder | 1.43 (0.80, 2.57) | 1.25 (0.63, 2.46) | 1.70 (0.93, 3.11) | 1.26 (0.89, 1.77) | 1.08 (0.72, 1.62) | 1.19 (0.81, 1.74) |
| **Substance use** |  |  |  |  |  |  |
| Problematic alcohol use | 1.33 (0.75, 2.36) | 2.42 (1.23, 4.75) | 1.15 (0.63, 2.11) | 0.97 (0.46, 2.04) | 1.39 (0.58, 3.35) | 0.83 (0.37, 1.90) |
| Problematic drug use | 1.26 (0.71, 2.24) | 1.88 (0.96, 3.70) | 0.85 (0.47, 1.54) | 1.09 (0.51, 2.34) | 1.15 (0.47, 2.81) | 0.55 (0.24, 1.28) |
| Current daily smoker | 0.97 (0.52, 1.79) | 0.95 (0.47, 1.94) | 0.67 (0.36, 1.26) | 0.73 (0.33, 1.63) | 0.50 (0.19, 1.27) | 0.47 (0.20, 1.13) |
| Enabling Factors |  |  |  |  |  |  |
| **Regular source of care**  Yes | 2.85 (1.57, 5.15) | 3.97 (1.92, 8.23) | 2.28 (1.23, 4.22) | 2.33 (1.20, 4.52) | 2.91 (1.28, 6.60) | 1.35 (0.64, 2.86) |
| **Perceived barrier to care**  *(past 6 to 12 months)* | 1.18 (0.66, 2.13) | 1.28 (0.65, 2.52) | 0.91 (0.49, 1.69) | 0.95 (0.48, 1.91) | 0.98 (0.43, 2.23) | 0.67 (0.31, 1.47) |
| **Food insecurity**  *(not enough food)* | 1.39 (0.78, 2.49) | 1.38 (0.70, 2.71) | 1.12 (0.61, 2.05) | 1.60 (0.81, 3.17) | 1.45 (0.65, 3.24) | 1.48 (0.69, 3.16) |
| **Acute mental health care**  *(past 12 months)* | 4.47 (2.09, 9.59) | 12.00 (5.13, 28.08) | 23.19 (10.31, 52. 17) | 3.64 (1.60, 8.27) | 8.26 (3.28, 20.82) | 17.00 (7.04, 41.04) |
| **Acute non-mental health care** *(past 12 months)* | 2.30 (1.28, 4.13) | 5.11 (2.50, 10.42) | 6.64 (3.50, 12.63) | 1.73 (0.88, 3.39) | 2.88 (1.27, 6.53) | 4.67 (2.17, 10.05) |
| Need Factors |  |  |  |  |  |  |
| **Perceived general health** |  |  |  |  |  |  |
| Fair | 1.02 (0.54, 1.94) | 0.75 (0.35, 1.59) | 0.71 (0.36, 1.41) | 0.99 (0.61, 1.59) | 0.82 (0.46, 1.46) | 0.75 (0.44, 1.28) |
| Poor | 1.09 (0.48, 2.45) | 0.98 (0.39, 2.49) | 1.27 (0.56, 2.91) | 0.84 (0.47, 1.52) | 0.84 (0.42, 1.67) | 1.15 (0.60, 2.19) |
| **Diagnosed conditions**  *(administrative records)* |  |  |  |  |  |  |
| 1 | 1.62 (0.80, 3.27) | 2.05 (0.91, 4.60) | 2.25 (1.08, 4.65) | 1.08 (0.58, 1.99) | 0.97 (0.49, 1.93) | 1.04 (0.54, 1.99) |
| 2+ | 2.05 (0.66, 6.39) | 4.17 (1.25, 13.92) | 4.66 (1.50, 14.45) | 1.20 (0.53, 2.71) | 1.72 (0.71, 4.16) | 1.80 (0.77, 4.20) |
| **Self-reported conditions**  *(survey data)* |  |  |  |  |  |  |
| 1 | 1.58 (0.81, 3.09) | 1.27 (0.56, 2.84) | 1.20 (0.59, 2.45) | - | - | - |
| 2+ | 1.47 (0.67, 3.22) | 2.28 (0.95, 5.44) | 1.63 (0.73, 3.64) | - | - | - |

***Supplemental Table S3:*** Fully adjusted and imputed odds ratio estimates for the associations between predisposing, enabling, and need factors and higher cost healthcare use for the general homeless cohort (Health and Housing in Transition study).

| Study | Odds Ratio (95%CI) | | | | | |
| --- | --- | --- | --- | --- | --- | --- |
|  | Age-adjusted | | | Fully adjusted | | |
| Cost category | Top 11-50%  (n=219) | Top 6-10%  (n=83) | Top 5%  (n=160) | Top 11-50%  (n=279) | Top 6-10%  (n=79) | Top 5%  (n=108) |
| Predisposing Factors |  |  |  |  |  |  |
| **Age**  *(per 1 year)* | - | - | - | 1.00 (0.98, 1.03) | 1.00 (0.97, 1.04) | 1.02 (0.98, 1.05) |
| **Gender** ^a^  Female | 1.84 (1.19, 2.84) | 2.36 (1.33, 4.20) | 2.48 (1.47, 4.19) | 1.62 (0.96, 2.75) | 2.11 (1.02, 4.34) | 2.19 (1.10, 4.35) |
| **Marital Status**  Single, never married | 0.66 (0.44, 0.97) | 0.73 (0.42, 1.27) | 0.73 (0.44, 1.20) | 0.76 (0.60, 0.96) | 0.81 (0.57, 1.14) | 0.85 (0.62, 1.18) |
| **Race**  Black | 0.97 (0.56, 1.69) | 0.55 (0.23, 1.35) | 0.30 (0.11, 0.81) | 1.04 (0.66, 1.63) | 0.88 (0.42, 1.83) | 0.54 (0.24, 1.18) |
| Other racialized groups | 1.02 (0.65, 1.62) | 0.70 (0.35, 1.39) | 1.09 (0.63, 1.92) | 0.90 (0.61, 1.32) | 0.75 (0.40, 1.41) | 1.09 (0.61, 1.96) |
| **Place of Birth**  Outside Canada | 1.08 (0.68, 1.72) | 0.99 (0.51, 1.93) | 0.47 (0.23, 0.97) | - | - | - |
| **Education**  Graduated high school | 0.75 (0.51, 1.10) | 0.75 (0.44, 1.28) | 0.62 (0.38, 1.00) | 0.75 (0.47, 1.20) | 0.86 (0.44, 1.65) | 0.74 (0.39, 1.38) |
| **Employment**  Currently employed | 0.56 (0.32, 0.99) | 0.62 (0.27, 1.42) | 0.27 (0.10, 0.72) | 0.67 (0.34, 1.31) | 0.88 (0.32, 2.45) | 0.46 (0.14, 1.52) |
| **Criminal behavior**  *(past 6 to 12 months)* | 1.47 (0.99, 2.19) | 1.58 (0.91, 2.74) | 1.76 (1.07, 2.90) | 1.02 (0.62, 1.68) | 0.96 (0.48, 1.93) | 1.03 (0.53, 2.01) |
| **Victimization**  *(past 6 to 12 months)* | 1.76 (1.17, 2.63) | 1.68 (0.96, 2.94) | 2.16 (1.30, 3.58) | 1.39 (0.85, 2.29) | 1.02 (0.50, 2.06) | 1.25 (0.64, 2.42) |
| **Housing Status**  Homeless | 0.71 (0.49, 1.03) | 0.66 (0.39, 1.11) | 0.72 (0.45, 1.17) | 0.67 (0.43, 1.05) | 0.70 (0.36, 1.34) | 0.61 (0.33, 1.13) |
| **Duration of homelessness**  (per 1-year increase) | 0.99 (0.96, 1.03) | 0.98 (0.94, 1.03) | 1.02 (0.99, 1.06) | 0.98 (0.95, 1.02) | 0.96 (0.91, 1.01) | 1.00 (0.95, 1.05) |
| ≥ 2 years spent homeless | 1.03 (0.71, 1.49) | 0.90 (0.53, 1.53) | 1.15 (0.71, 1.86) | - | - | - |
| **Diagnosed of mental illness** |  |  |  |  |  |  |
| Psychotic disorder | 0.93 (0.33, 2.58) | 1.74 (0.57, 5.27) | 2.59 (0.90, 7.45) | 1.84 (0.95, 3.59) | 3.98 (1.84, 8.64) | 3.20 (1.52, 6.73) |
| Other disorder *(reference)* |  |  |  |  |  |  |
| No disorder | 0.17 (0.09, 0.29) | 0.11 (0.05. 0.23) | 0.11 (0.06, 0.22) | 0.37 (0.25, 0.56) | 0.24 (0.14, 0.40) | 0.29 (0.17, 0.48) |
| **Substance use** |  |  |  |  |  |  |
| Problematic alcohol use | 1.65 (0.99, 2.73) | 1.85 (0.95, 3.61) | 2.44 (1.35, 4.42) | 0.89 (0.47, 1.71) | 1.12 (0.47, 2.67) | 0.77 (0.34, 1.78) |
| Problematic drug use | 3.29 (2.06, 5.26) | 7.77 (4.22, 14.32) | 7.05 (4.01, 12.39) | 2.25 (1.28, 3.94) | 5.38 (2.50, 11.55) | 3.62 (1.75, 7.46) |
| Current daily smoker | 0.97 (0.63, 1.50) | 1.00 (0.54, 1.86) | 1.10 (0.63, 1.92) | 1.04 (0.60, 1.78) | 0.93 (0.43, 2.03) | 1.16 (0.55, 2.45) |
| Enabling Factors |  |  |  |  |  |  |
| **Regular source of care**  Yes | 2.08 (1.43, 3.03) | 4.15 (2.31, 7.46) | 4.56 (2.67, 7.78) | 1.60 (1.02, 2.50) | 3.00 (1.50, 5.99) | 3.17 (1.63, 6.15) |
| **Perceived barrier to care**  *(past 6 to 12 months)* | 1.87 (1.24, 2.82) | 2.58 (1.48, 4.48) | 1.77 (1.06, 2.96) | 1.63 (1.00, 2.63) | 1.99 (1.02, 3.88) | 1.37 (0.71, 2.65) |
| **Food insecurity**  *(not enough food)* | 0.88 (0.59, 1.31) | 1.65 (0.96, 2.84) | 1.16 (0.70, 1.92) | 0.77 (0.47, 1.25) | 1.49 (0.76, 2.93) | 0.79 (0.41, 1.54) |
| **Acute mental health care**  *(past 12 months)* | 4.43 (2.12, 9.27) | 6.42 (2.75, 14.99) | 21.55 (9.92, 46.79) | 1.97 (0.80, 4.87) | 1.21 (0.40, 3.67) | 6.49 (2.35, 17.96) |
| **Acute non-mental health care** *(past 12 months)* | 2.89 (1.93, 4.33) | 6.03 (3.40, 10.69) | 5.34 (3.19, 8.92) | 1.86 (1.16, 2.99) | 3.53 (1.78, 7.01) | 2.25 (1.18, 4.30) |
| Need Factors |  |  |  |  |  |  |
| **Perceived general health** |  |  |  |  |  |  |
| Fair | 1.41 (0.91, 2.17) | 2.91 (1.61, 5.25) | 2.02 (1.15, 3.54) | 1.01 (0.69, 1.48) | 1.35 (0.82, 2.23) | 1.00 (0.62, 1.61) |
| Poor | 1.85 (0.95, 3.60) | 3.35 (1.41, 7.92) | 5.15 (2.48, 10.66) | 1.16 (0.69, 1.95) | 1.19 (0.61, 2.32) | 1.72 (0.93, 3.18) |
| **Diagnosed conditions**  *(administrative records)* |  |  |  |  |  |  |
| 1 | 3.18 (1.93, 5.26) | 6.08 (3.15, 11.74) | 3.82 (2.01, 7.25) | 1.43 (0.92, 2.22) | 1.46 (0.85, 2.51) | 0.88 (0.51, 1.50) |
| 2+ | 3.62 (1.58, 8.28) | 11.31 (4.33, 29.54) | 16.50 (6.96, 39.14) | 1.33 (0.73, 2.44) | 2.30 (1.14, 4.67) | 3.17 (1.64, 6.12) |
| **Self-reported conditions**  *(survey data)* |  |  |  |  |  |  |
| 1 | 1.94 (1.26, 2.99) | 2.32 (1.20, 4.48) | 1.65 (0.88, 3.08) | - | - | - |
| 2+ | 2.88 (1.50, 5.51) | 6.38 (3.17, 12.85) | 7.27 (3.88, 13.62) | - | - | - |

***Supplemental Table S4:*** Multinomial odds ratio estimates for the associations between predisposing, enabling, and need factors and healthcare expenditure categories for the At Home / Chez Soi participants, adjusting for the Housing First intervention (n=525).

| Cost category | Odds Ratio (95%CI) | | |
| --- | --- | --- | --- |
|  | Top 11-50% (n=219) | Top 6-10% (n=83) | Top 5% (n=160) |
| Predisposing Factors |  |  |  |
| **Age**  Per 1 year | 1.00 (0.98, 1.03) | 1.01 (0.98, 1.04) | 1.01 (0.98, 1.03) |
| **Age Group**  18 to 34 *(reference)*  35 to 49  50+ | -  1.81 (0.94, 3.49)  1.17 (0.56, 2.45) | -  2.09 (0.95, 4.59)  1.57 (0.65, 3.80) | -  1.73 (0.88, 3.42)  1.08 (0.50, 2.33) |
| **Gender** ^a^  Female | 1.65 (0.85, 3.18) | 1.18 (0.54, 2.56) | 1.67 (0.84, 3.29) |
| **Marital Status**  Single, never married | 0.71 (0.37, 1.36) | 0.55 (0.26, 1.14) | 0.92 (0.46, 1.83) |
| **Race**  Black | 0.65 (0.31, 1.34) | 0.45 (0.20, 1.03) | 0.42 (0.20, 0.89) |
| Other racialized groups | 0.59 (0.29, 1.22) | 0.32 (0.14, 0.75) | 0.39 (0.18, 0.81) |
| **Born in Canada**  No | 1.26 (0.71, 2.24) | 0.71 (0.36, 1.39) | 0.86 (0.47, 1.58) |
| **Education**  Graduated high school | 0.79 (0.44, 1.40) | 0.92 (0.47, 1.79) | 1.14 (0.62, 2.08) |
| **Employment**  Currently employed | 0.30 (0.10, 0.94) | 0.34 (0.08, 1.43) | 0.31 (0.09, 1.06) |
| **Housing Status**  Homeless | 0.69 (0.23, 2.10) | 0.64 (0.18, 2.22) | 1.50 (0.42, 5.33) |
| **Duration of homelessness**  Per 1-year increase | 0.98 (0.94, 1.03) | 1.00 (0.95, 1.05) | 0.98 (0.93, 1.03) |
| ≥ 2 years vs <2 years | 1.04 (0.58, 1.86) | 0.75 (0.38, 1.48) | 0.89 (0.49, 1.64) |
| **Criminal behavior**  *(past 6 to 12 months)* | 1.88 (0.98, 3.58) | 2.86 (1.38, 5.91) | 2.06 (1.05, 4.01) |
| **Victimization**  *(past 6 to 12 months)* | 2.03 (1.03, 3.99) | 2.02 (0.94, 4.34) | 2.35 (1.17, 4.72) |
| **Diagnosed of mental illness**  Psychotic disorder | 1.45 (0.81, 2.60) | 1.23 (0.62, 2.43) | 1.70 (0.93, 3.12) |
| **Substance use** |  |  |  |
| Problematic alcohol use | 1.31 (0.74, 2.33) | 2.34 (1.20, 4.58) | 1.14 (0.63, 2.07) |
| Problematic drug use | 1.23 (0.70, 2.15) | 1.75 (0.90, 3.40) | 0.83 (0.46, 1.49) |
| Current daily smoker | 0.97 (0.53, 1.80) | 0.94 (0.46, 1.93) | 0.67 (0.36, 1.27) |
| Enabling Factors |  |  |  |
| **Regular source of care**  Yes | 2.87 (1.59, 5.18) | 4.06 (1.97, 8.35) | 2.32 (1.26, 4.26) |
| **Perceived barrier to care**  *(past 6 to 12 months)* | 1.16 (0.65, 2.09) | 1.26 (0.64, 2.49) | 0.90 (0.48, 1.66) |
| **Food insecurity**  *(not enough food)* | 1.36 (0.77, 2.42) | 1.32 (0.68, 2.58) | 1.09 (0.60, 2.00) |
| **Acute mental health care**  *(past 12 months)* | 4.37 (2.05, 9.31) | 10.70 (4.64, 24.72) | 21.08 (9.48, 46.88) |
| **Acute non-mental health care** *(past 12 months)* | 2.30 (1.28, 4.12) | 4.89 (2.41, 9.92) | 6.50 (3.43, 12.32) |
| Need Factors |  |  |  |
| **Perceived general health** |  |  |  |
| Fair | 1.03 (0.54, 1.94) | 0.77 (0.36, 1.63) | 0.72 (0.37, 1.42) |
| Poor | 1.07 (0.48, 2.41) | 1.01 (0.40, 2.57) | 1.27 (0.56, 2.90) |
| **Diagnosed conditions**  *(administrative records)* |  |  |  |
| 1 | 1.62 (0.80, 3.27) | 2.05 (0.91, 4.60) | 2.24 (1.08, 4.65) |
| 2+ | 2.05 (0.68, 6.23) | 4.28 (1.33, 13.78) | 4.45 (1.48, 13.40) |
| **Self-reported conditions**  *(survey data)* |  |  |  |
| 1 | 1.57 (0.80, 3.07) | 1.27 (0.57, 2.86) | 1.20 (0.59, 2.44) |
| 2+ | 1.51 (0.70, 3.25) | 2.38 (1.02, 5.57) | 1.68 (0.76, 3.69) |
